# Suspiciousness

**Published:** 1878-06

**Authors:** 


### SUSPICIOUSNESS.

One of the most laughable and amusing occupations of a publisher is reading le ters from country folk in reference to orders for books, magazines, newspapers, &c. The extreme readiness to jump at the idea that somebody wants to cheat them out of the worth of two cents is amazing. They hear so much of the rascalities of large cities that they become afraid of their own shadow; being entirely oblivious of the fact that four times out of five the thousand and one little meannesses of which they read have been perpetrated by their own friends and neighbors and relations, who have come to New York to try their hands at all sorts of ill doings, but have'nt sense enough to keep themselves from being found out.

